# Preparation of Near-Infrared/Photoacoustic Dual-Mode Imaging and Photothermal/Chemo Synergistic Theranostic Nanoparticles and Their Imaging and Treating of Hepatic Carcinoma

**DOI:** 10.3389/fonc.2021.750807

**Published:** 2021-09-17

**Authors:** Yun Zhou, Bixia Lin, Kai Li, Yufeng Zhao, Zhuo Sun, Chenchen He, Rajiv Kumar Jha

**Affiliations:** ^1^College of Clinical Medicine, Xi'an Medical University, Xi’an, China; ^2^College of Pharmacy, Xi'an Medical University, Xi’an, China; ^3^Department of Basic Medical Science, Xi’an Medical University, Xi’an, China; ^4^Department of Radiation Oncology, First Affiliated Hospital of Xi’an Jiaotong University, Xi’an, China

**Keywords:** hepatic carcinoma, theranostic, nanocarrier, dual-mode imaging, synergistic therapy

## Abstract

At present, the clinical diagnosis of and treatment methods for hepatic carcinoma still fail to fully meet the needs of patients. The integrated theranostic system, in which functional materials are used to load different active molecules, created a new developmental direction for the combination treatment of hepatic carcinoma, realizing the synchronization of diagnosis and treatment. In this study, polydopamine (PDA), which has the functions of self-assembly, encapsulation, photothermal conversion, and photoacoustic interaction, was used as the carrier material. The IR780, a near-infrared fluorescence imaging (NIFI), photoacoustic imaging (PAI), and photothermal therapy (PTT) agent, and paclitaxel (PTX), a broad-spectrum chemotherapy drug, were selected to build the NIF/PA dual-mode imaging and PTT/chemo synergistic theranostic nanoparticles (DIST NPs). The DIST NPs have a 103.4 ± 13.3 nm particle size, a weak negative charge on the surface, good colloidal stability, slow and controlled drug release, and high photothermal conversion ability. The experiments results showed that the DIST NPs have a long circulation *in vivo*, high bioavailability, high biocompatibility, and low effective dose. DIST NPs showed an excellent NIFI/PAI dual-mode imaging and significant synergistic antitumor effect in hepatic carcinoma models. DIST NPs met the initial design requirements. A set of fast and low-cost preparation methods was established. This study provides an experimental basis for the development of new clinical theranostic methods for hepatic carcinoma.

## Introduction

According to statistics, more than 900,000 new cases and more than 830,000 deaths from hepatic carcinoma were recorded worldwide in 2020. Among them, China accounts for 45.3% of new cases and 47.1% of deaths of the world data (1–3). At present, hepatic carcinoma is mainly diagnosed by imaging, identification factors test, and pathological analysis. However, the effect is not satisfactory. When patients are diagnosed with hepatic carcinoma, most of them have reached the middle and advanced stage, which is difficult to treat with surgical treatment. The patients can only focus on non-surgical local treatment and systematic treatment (4, 5). Even those who can undergo surgical resection still face postoperative recurrence and metastasis (6). Medicine therapy has made some progress in recent years, and the median survival rate of patients has improved (7, 8). Nevertheless, the overall treatment effect is still far from expected. Therefore, it is urgent to develop a new diagnosis and treatment technology for hepatic carcinoma. Because of the characteristics of tumor, such as heterogeneity, diversity, and complexity, a single treatment or diagnosis method often fails to ensure comprehensiveness. Therefore, theranostics, the integration of diagnosis and treatment, of which the purpose is to realize the effects of diagnosis adjuvant treatment and simultaneous diagnosis, came into being. Theranostics has prominent advantages in clinical tumor treatment (9).

In the integrated theranostics system, various diagnosis and treatment functions must be integrated under reasonable technology, supporting each other and working together. PAI is a new medical imaging technology combining ultrasound and optics. Its main mechanism is that the agent absorbs external energy, resulting in local heat, thermoelastic expansion, and transient acoustic signals. Various biological tissues have different absorption coefficients. After measurement and reconstruction, the photoacoustic data of the corresponding tissue was converted into images (10, 11). PAI has the advantages of high spatial resolution, large imaging depth, non-ionized radiation, and low cost. In addition, it can also show the physiological tissue structure, function, metabolism, and other characteristics and provide more information on the images through endogenous or exogenous molecular probes. The PAI has opened up new ways for clinical imaging of different purposes, including brain function imaging, tumor monitoring, and skin lesion detection (12, 13). The commonly reported molecular probes include hemoglobin, melanin, lipids, and various compounds with photothermal effects (14, 15). As a molecular probe that can be used for PAI, NIF dyes with strong fluorescence emission characteristics have natural advantages for the PAI and NIF dual-mode imaging. NIFI is a non-invasive, fast, and simple imaging method. Its detection wavelength was longer than 700 nm, which has an excellent tissue penetration, and a weak tissue absorption. It is an ideal method in optical imaging (16). IR780, a lipophilic NIF dye, has been widely used in various *in vivo* imaging studies (17). In addition, it also has a strong photothermal conversion performance. The dye could perform PAI and NIFI simultaneously. However, it is almost impossible to obtain a satisfactory effect based solely on a function of IR780. Reasonable utilization of diversified materials is bound to be the primary selection. Melanin, another PAI contrast agent, also attracted much attention. Dopamine, a biological neurotransmitter, can form dopamine quinone structures in an alkaline environment, polymerizing into PDA, which is also known as artificial melanin (18–21). In addition to PAI function, PDA is also used in nano drug delivery systems (22–24). Therefore, the combination of PDA and IR780 can meet the requirements of NIFI and PAI dual-mode imaging of hepatic carcinoma. PDA, like IR780, also has a photothermal conversion effect, further providing the therapeutic effect. Overall, this combination fully meets the needs of establishing an integrated theranostics system.

PTT is a novel tumor treatment technology. The photothermal conversion agent accumulated in the tumor generates heat energy under an external light source irradiation, causing tumor cell damage and necrosis and tumor tissue thermal ablation. The action time of PTT is short, but the therapeutic effect is remarkable. After PTT, the trauma is minor, and the side effects are insignificant (25, 26). However, PPT is not a perfect treatment for tumors. This is because, the inherent biological complexity and dynamic changes of tumors make PTT difficult to completely ablate tumor tissues, weakening the effect of treatment. If another method could be combined with PTT, and remedy the defect, the treatment will obtain a better effect. In the PTT treatment process, it can also promote the permeation and cell absorption of chemotherapeutic drugs, improving chemotherapy. The advantage could overcome the defect of PTT. Therefore, PTT and chemotherapy have a synergistic therapeutic effect (27, 28). Although having apparent advantages, the traditional combination therapy is still not capable of a synergistic tumor therapy. The reason is that there are substantial pharmacokinetic differences between different drugs or active molecules. It is challenging to achieve synchronous and sufficient accumulation in complex tumor tissues (29, 30). Therefore, the key to solving this problem is to deliver and accumulate various therapeutic molecules into the tumor synchronously.

Nano drug carriers bring new ideas to solve these problems. They can improve the bioavailability of therapeutic molecules, eliminate the difference in metabolic kinetics, and improve tumor tissue accumulation to overcome the limitations of a direct administration (31–33). Based on this, we intend to build a nanocarrier by PDA, IR780, and PTX to integrate NIFI/PAI dual-mode imaging and PTT/chemotherapy functions in one system through the nanocarrier technology to develop a new system for the theranostics of hepatic carcinoma. In this system, PDA is the essential material. Firstly, PDA was used as a coating material with a good loading performance. PTX and IR780 that contain aromatic ring structures can be loaded in PDA nanocarriers through π-π stacking and hydrogen bonding effects (34, 35). Secondly, as an artificial melanin, PDA also has good PAI and PTT abilities. In addition, the nanocarriers formed by PDA can also realize the ultra-long circulation and pH-responsive release *in vivo* (24, 36). These properties ensure that the prepared nanocarriers can effectively integrate a variety of functions. More importantly, the preparation process is straightforward, fast, and cheap. It can provide an experimental basis for developing new clinical treatment methods for hepatic carcinoma.

## Materials and Methods

### Materials

Dopamine hydrochloride and PTX were purchased from Sigma Aldrich Co., Ltd. Coumarin 6, IR780, and Pluronic F127 were purchased from Aladdin Co., Ltd. CCK-8 kit was purchased from Topscience Inc. DAPI kit was purchased from Biotech Ltd. The apoptosis detection kit was purchased from Beyotime Inc. Biotechnology. IL-6 detection kit and TNF-α test kit were purchased from Wuhan Servicebio Ltd. The other chemical reagents were purchased from Sinopharm Lnc. The Huh-7, BEL-7402, HepG-2, and HL-7702 Cell lines were all derived from ATCC. The BALB/C mice and BALB/c-nu/nu mice were purchased from Beijing HFK Biotechnology Ltd. The CB-17 SCID mouse was purchased from Charles River (Beijing) Experimental Animal Technology Ltd. Unless otherwise stated, all the chemicals and reagents were of analytical grade and used as received.

### Preparation of the Dual-Mode Imaging and PTT/Chemo Synergistic Theranostic Nanoparticles

In a round bottom flask, 4 ml of Tris HCl (0.5 mg/ml, water) was placed. A total of 1 mg of PTX and 4 mg of Pluronic F127 was dissolved in 200 μl of DMSO. The solution was added dropwise to the Tris HCl solution under stirring (800 rpm). And then, 100 μL of IR780 (20 mg/ml, DMSO) was added to the solution. The solution was stirred continuously for 20 min before being treated by a 150 W ultrasound for 5 min. After that, 1 ml of dopamine hydrochloride solution (10 mg/ml, water) was slowly added to the solution above. After stirring for 5 min, the solution was sealed and rotated in the dark for more than 72 h. Then, the solvent and soluble impurities were removed by dialysis against the Tris HCl solution, and the precipitations were removed by low-speed centrifugation to obtain the final DIST NPs solution.

### Dual-Mode Imaging and PTT/Chemo Synergistic Theranostic Nanoparticles Performance Characterization

The particle size and zeta-potential distributions of DIST NPs were detected by the dynamic light scattering method. The morphology of DIST NPs was observed by a transmission electron microscope (TEM, Tecnai G2 F20 U-TWIN, FEI, USA). The DIST NPs were decomposed in an acidic environment to release PTX and IR780. Chloroform was used to extract the two substances. The concentrations of PTX and IR780 were detected by HPLC and fluorescence spectrophotometry, respectively. Then, the encapsulation rate and drug loading capacity of DIST NPs were calculated. The particle size changes of DIST NPs in PBS, complete medium, and fetal bovine serum were continuously measured to determine their colloidal stability in different physiological environments. The release degree of DIST NPs under different pH and temperature conditions were detected by the dialysis method. In evaluation of the photothermal conversion, different parameters, such as concentrations and irradiation time, were set, and temperature was measured by a thermal imager (E4, Teledyne FLIR, USA). The wavelength of the laser is 808 nm. *In vivo* evaluation of thermal conversion was performed in the BALB/c mouse model. The mice were depilated by a cream for a more effective observation. The DIST NPs was subcutaneously injected in the crotch of mouse. Subsequently, the 808-nm laser was used to irradiate the injection area. The infrared thermal imager was employed to measure the temperature.

### Dual-Mode Imaging and PTT/Chemo Synergistic Theranostic Nanoparticles *In Vitro* Delivery

After referring to a variety of preparation protocols, DIST NPs were labeled with coumarin 6 to have a green fluorescence (24, 36). BEL-7402 cells were then planted in 3.5-cm glass-bottom dishes (1 × 10^5^ cells/dish) and cultured at 37°C and 5% CO_2_ for 48 h. Then, the cells were incubated with fluorescence-labeled DIST NPs and observed at different time points by a laser scanning confocal microscope (TCS SPT, Leica, Germany). In order to determine the internalization effect of DIST NPs, the cells were treated with sodium azide, an endocytosis inhibitor, and incubated with DIST NPs for confocal observation and comparison.

### Tumor Inhibition Effect of the Dual-Mode Imaging and PTT/Chemo Synergistic Theranostic Nanoparticles *In Vitro*

The cell inhibition of DIST NPs *in vitro* was tested by CCK-8 assay. The hepatic carcinoma cell lines (Huh-7, BEL-7402, HepG2) and a normal hepatic cell line (HL-7702) in their logarithmic growth phase were inoculated into 96-well plates (8 × 10^3^ cells/well). Then, the DIST NPs, PTX, IR780, and PDA were added, respectively. The cells were cultured at 37°C and 5% CO_2_. After the cells in the control wells reached 90% confluence, the obsolete medium in the well was removed, and the colorless cultured medium containing 10% CCK-8 was added. The cells were incubated for 2 h before being put it into a plate reader (ELx800, BioTek, USA) to measure the absorbance at 450 nm each well for the cell viability calculation.

The combined treatment of PTT-chemotherapy was evaluated by CCK-8 assay, colony formation test, and flow cytometry method. The cell lines were Huh-7, BEL-7402, and HepG2. In the CCK-8 assay, the cells were inoculated into 96-well plates (4 × 10^4^/well) at 37°C and 5% CO_2_ for 24 h. After the DIST NPs, PTX, IR780, and PDA were added, the cells were irradiated with an 808-nm laser, and then incubated for 6 h. The culture medium was replaced by the colorless culture medium containing CCK-8. The cells were incubated for 2 h before being put into a plate reader to measure the absorbance at 450 nm for the cell viability calculation. In the colony formation test, 500 μl of cell suspension (2,000 cells/ml) was added into a 1.5-ml centrifuge tube. The sample was added, and laser irradiation was performed, and then the cell suspension was transferred to a 6-cm plate and cultured for five days. After that, the colony was stained for observation. After the cells were treated with PTT, chemotherapy, and combination therapy, the cells were stained with a cell apoptosis kit and tested by a flow cytometer (Accuri C6 Plus, BD, USA).

### *In Vivo* Toxicity of the Dual-Mode Imaging and PTT/Chemo Synergistic Theranostic Nanoparticles

DIST NPs are supposed to be injected intravenously, and their circulation time should be long in the body. Therefore, it is necessary to evaluate whether the DIST NPs and their components can cause hemolysis. A 2% erythrocyte suspension was prepared and divided into groups. Then, the DIST NPs and their main components were added to the test groups, respectively. The water, saline, and 0.1% Triton X-100 were added to the control groups, respectively. The red blood cells were incubated at 37°C for 2 h. The supernatant was replaced by the same amount of water after centrifugation before another 4 h of incubation. The absorbance wavelength at 540 nm in each group was measured by a microplate reader, and the hemolysis rate was calculated.

In the acute toxicity test, 40 BALB/c mice, half male and half female, weighing about 20 g, were selected and randomly divided into four groups. DIST NPs, PTX, IR780, and PDA were intravenously injected into the mice, respectively. All the samples injected were adjusted to the equivalent PTX concentration of 5 mg/kg or IR780 concentration of 10 mg/kg. The mice were prohibited from food and water 6 h before injection and were resumed for 2 h after the injection. The mice were observed continuously for 14 d. The symptoms were recorded, and the survival rate was calculated. Finally, the surviving mice were euthanized. The main organs were taken for pathological analysis.

The levels of interleukin-6 (IL-6) and tumor necrosis factor (TNF-α) in the blood of mice were tested by the ELISA kit to evaluate whether the DIST NPs can cause a systemic inflammatory reaction *in vivo*. The grouping is the same as the acute toxicity test except that there were four mice in each group. The samples were injected through the tail vein, and the blood samples were collected 24 h after injection.

All the animal experiments in this study were approved by the Laboratory Animal Administration Committee of Xi’an Medical University. The protocols for the animal experiments followed the Guidelines for the Use and Care of Experimental Animals at Xi’an Medical University. The Animal Ethics Approved Document Number is XY-AUC-2020-341.

### *In Vivo* Hepatic Carcinoma Model

Four-week-old female BALB/c-nu/nu mice were reared in an SPF room for five days for acclimation. Then, 150 μl of the BEL-7402 cell suspension (1 × 10^6^/ml) was injected into the crotch of each mouse. The tumor-bearing mice were used for subsequent experiments after their tumors grew to a suitable volume.

The orthotopic hepatic carcinoma model was established by CB-17 SCID mice. The 6-week-old female mice were reared in the SPF room for five days. After their abdominal hairs were removed, the mice were given general anesthesia through the inhalation of isoflurane and were fixed on the operating platform. A vertical incision with a length of 1 cm was cut below 1 cm of the xiphoid process. The peritoneum was cut, and the wound was expanded with a retractor. A small wound with 3–5 mm length and 1–2 mm depth was gently drawn on the surface of the liver. The fresh tumor tissue obtained from the subcutaneous tumor was planted in the wound. The wound on the liver was bonded with surgical glue. After the major wound was disinfected and sutured, lidocaine was injected subcutaneously by the side of the wound for analgesia. The mice were placed in a clean cage for observation and were given high nutritional feed. Their wounds were disinfected every day. After their wounds recovered, the mice were continually checked by abdominal touch. Once the clear indurations were found, the mice were used for subsequent experiments.

### Dual-Mode Imaging and PTT/Chemo Synergistic Theranostic Nanoparticles *In Vivo* Imaging

In the NIFI investigation, two orthotopic hepatic carcinoma model mice were injected with the DIST NPs and the same concentration of IR780 solution, respectively. The distribution of fluorescent signals in mice was continuously observed by the IVIS imaging system (PE, USA). The excitation and emission wavelengths were 780 nm and 845 nm, respectively. After the observation, the mice were euthanized, and the main organs and tumor tissues were taken out to analyze the distribution of the DIST NPs in the tissues.

In the PAI experiments, two subcutaneous tumor-bearing mice were injected with the DIST NPs and the same concentration of IR780 solution, respectively. The coupling agent was evenly coated on the mice. Wrapped with film and maintained with anesthesia, the mice were fixed in the supporting device and placed in the water tank for PAI. The excitation wavelength was 780 nm, and the emission range was 800–900 nm.

### Tumor Treatment of the Dual-Mode Imaging and PTT/Chemo Synergistic Theranostic Nanoparticles *In Vivo*

A total of 25 single side tumor-bearing mice were divided into five groups. The treatment methods were normal saline, IR780 + laser, PTX, IR780 + PTX + laser, and DIST NPs + laser. The dosage was calculated according to the concentrations of PTX and IR780, which were 1 mg/kg and 2 mg/kg, respectively. The dosage was determined in the pre-experiment, which could effectively demonstrate the difference of the treatment effect between the treatments. The ratio of IR780 and PTX causes the preparation craft of DIST NPs, that is 2:1. The administration was through intravenous injection. The laser irradiation time after injection was determined according to the experimental results of *in vivo* imaging. The irradiation wavelength, power, and time were 808 nm, 1 W/cm^2^, and 2 min, respectively. The whole treatment process was conducted twice a week. The size of tumors and the weight of mice were measured continually, and the tumor area was photographed. After 21 d, the mice were euthanized, and the tumor tissues were taken, weighed, and photographed.

### Statistical Analysis

The two-way ANOVA and t-test were used for statistical analysis. The software was GraphPad prism 5.0. The data of independently repeated experiments were presented as the mean values ± standard deviation (SD). A P-value less than 0.05 indicated a statistical difference.

## Results

### Characteristics and Performance of the Dual-Mode Imaging and PTT/Chemo Synergistic Theranostic Nanoparticles

**Figure 1:**
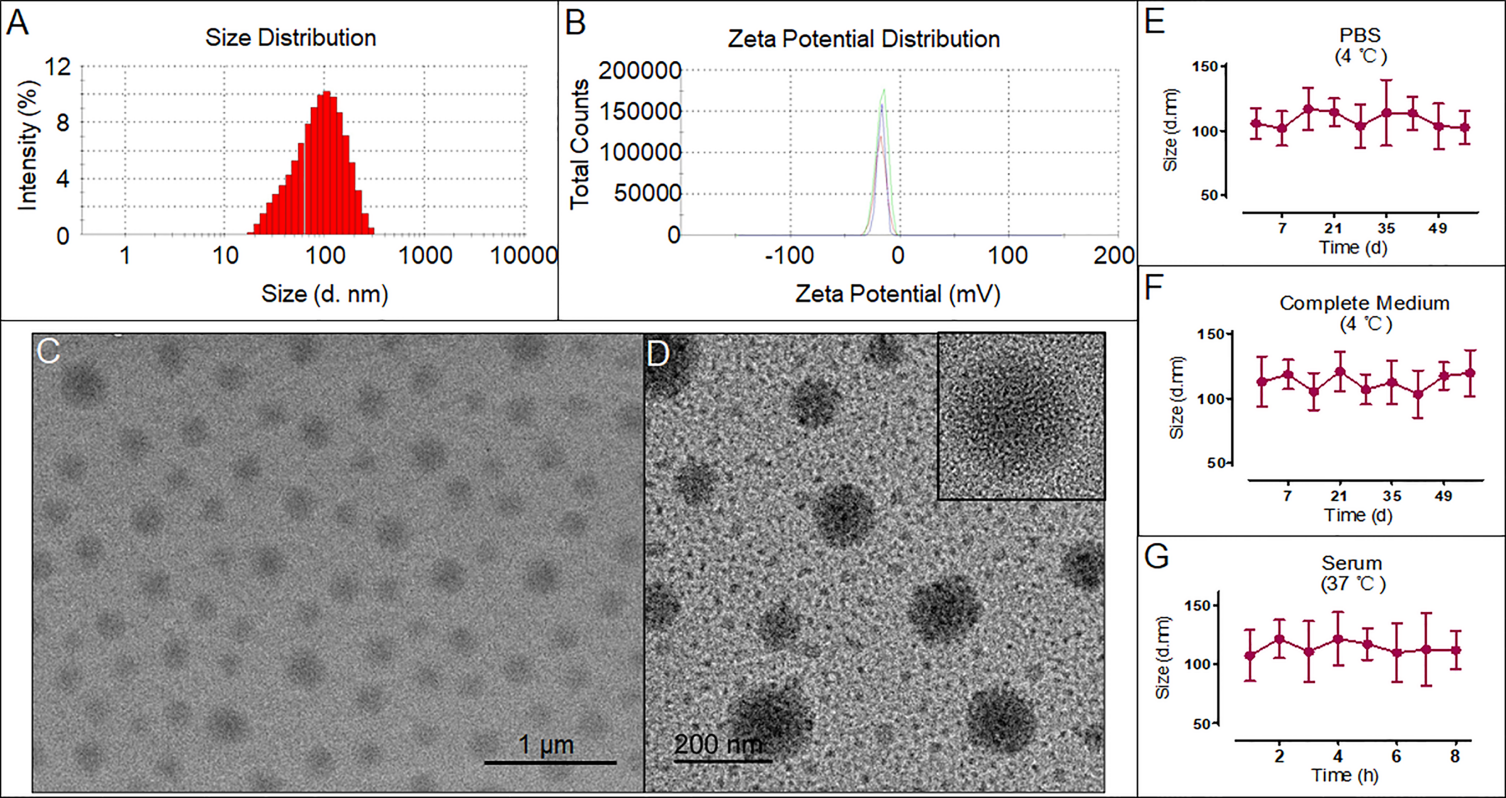
The characteristics and morphology of the DIST NPs. The size distribution **(A)** and zeta potential distribution **(B)** of the DIST NPs. The TEM **(C)** and high-resolution TEM **(D)** results of the DIST NPs. The colloid stability evaluated by the size change of the DIST NPs in various solvent and temperature environments. The quantitative experiment was repeated three times **(E–G)**.

As shown in **Figures 1A, B**, the size and surface potential distributions of the DIST NPs are 103.4 ± 13.3 nm and -16.7 ± 3.8 mV, respectively. The TEM results in **Figure 1C** show that the DIST NPs have a spherical shape, mono-dispersity, and uniform particle size. Further observation by high-resolution TEM shows that the DIST NPs are dense solid spheres (**Figure 1D**). The result preliminarily determined that the DIST NPs were successfully constructed. The overall particle size meets the requirements of effectively entering the tumor tissue through the enhanced permeability and retention (EPR) effect. Their surfaces are relatively smooth and negatively charged, indicating that they can effectively escape from the clearance by the reticuloendothelial system *in vivo* and are expected to maintain a long circulation time. The encapsulation rates of DIST NPs for PTX and IR780 are 93.4 ± 3.1% and 92.3 ± 4.8%, respectively. The drug loading capacities of the DIST NPs for PTX and IR780 were 12.7 ± 4.1% and 24.9 ± 5.8%, respectively. The stability evaluation results of the DIST NPs are shown in **Figures 1E–G**. The size of the DIST NPs did not change significantly under various simulated physiological environments, indicating a good colloidal stability of the DIST NPs.

**Figure 2:**
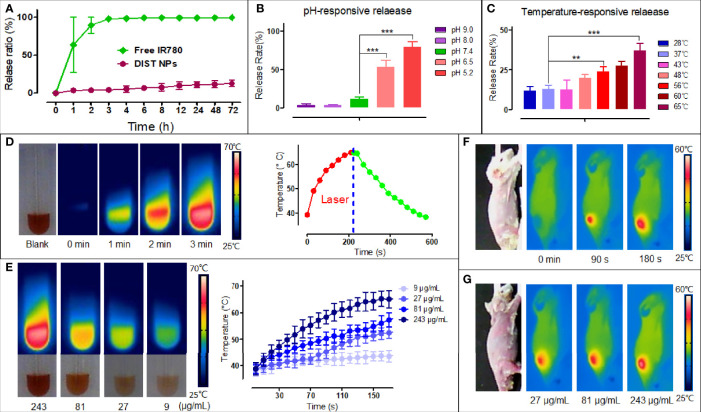
The releasing test results and photothermal properties of the DIST NPs. The IR780 releasing curve of the DIST NPs in the PBS solution with free IR780 as a control **(A)**. The releasing results of the DIST NPs in different pH environments. The pH 5.2 and 6.5 simulated the pH environments in the lysosome and tumor microenvironment, respectively **(B)**. The releasing degrees of the DIST NPs under different temperatures. The 65°C and 56°C simulated the maximum temperature that the DIST NPs can get under laser irradiations *in vivo* and *in vitro*, respectively **(C)**. The photothermal conversion results of the DIST NPs **(D)**. The temperature elevation of the DIST NPs with different concentrations **(E)**. The temperature elevation of the DIST NPs under the skin of mice. The temperature rise in different irradiation time **(F)**, and in different injection dose **(G)**. The quantitative experiment was repeated three times. The ** represents p < 0.01; and ***p < 0.001.

According to the *in vivo* delivery requirements, the DIST NPs should have long circulation and pH/temperature responding release abilities. **Figure 2A** shows the IR780 release curve of the DIST NPs in PBS at room temperature. Free IR780 exhibited an apparent burst release in which most of the IR780 was released within 2 h. However, only approximately 12% of IR780 in the DIST NPs was released in 72 h, indicating a good encapsulation stability of the DIST NPs. **Figure 2B** is the release curve of the DIST NPs under different pH environments. The release degree gradually increased with the decrease of the pH value. This scenario demonstrates that the DIST NPs can effectively release their loaded drugs under the acidic environment in tumor tissues and cells. **Figure 2C** shows the release curve of the DIST NPs at different temperatures. The temperature elevation promoted the release, indicating that the increase of temperature is also conducive to the drug release of the DIST NPs during PTT. **Figure 2D** shows the photothermal conversion results of the DIST NPs. With the same concentration, the temperature of the DIST NPs solution gradually increases with the extension of the irradiation time. The maximum temperature was 65°C. As shown in **Figure 2E**, there is a concentration-effect relationship between the concentration and temperature of the DIST NPs under the same irradiation power and time length. The 243 μg/ml DIST NPs had a maximum temperature of 64°C. The photothermal conversion effects *in vivo* are shown in **Figures 2F, G**. It can be seen that the DIST NPs can still absorb the laser energy and generate heat under the skin of mice. Their temperature reached more than 55°C in 3 min under irradiation. The temperature rise exhibited a positive relationship both with the concentration and irradiation time. These results indicate that the DIST NPs can be used for photothermal treatment *in vivo*.

### The Cell Delivery of the Dual-Mode Imaging and PTT/Chemo Synergistic Theranostic Nanoparticles

**Figure 3:**
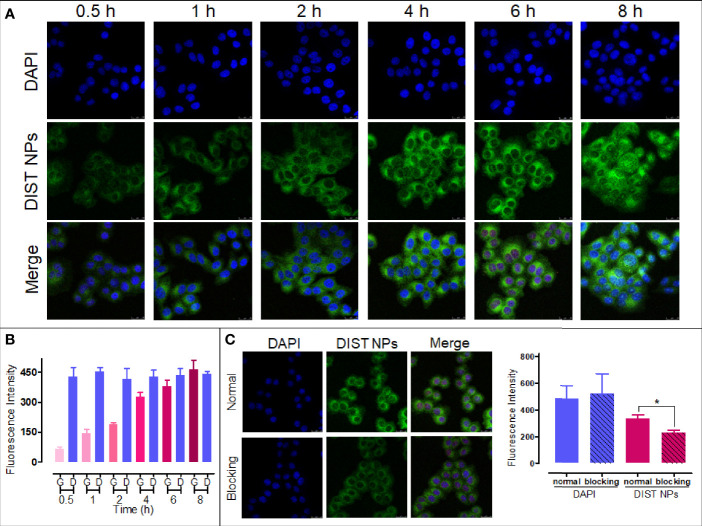
The cell endocytosis test results. The endocytosis effect **(A)** and fluorescence quantitative analytics **(B)** results of the DIST NPs. The endocytosis inhibition and fluorescence quantitative analytics results of the DIST NPs **(C)**. The data of signal quantitation were extracted at more than three areas. The * represents p < 0.05.

According to our previous study, DIST NPs were labeled with a green fluorescence for confocal microscopic observation (36). The results show that the DIST NPs can effectively enter the BEL-7402 cells. As shown in **Figure 3A**, with the extension of the incubation time, the intensity of intracellular fluorescent signal gradually increased, gathering in the cytoplasm. In the quantitative analysis of fluorescent signals (**Figure 3B**), the fluorescent intensity of the cytoplasm is gradually enhanced compared with that of DAPI in the nucleus. In order to prove that the DIST NPs entered the cells through endocytosis, sodium azide was used to pretreat the cells. As shown in **Figure 3C**, the fluorescent signal in the sodium azide-treated cells was significantly weaker than in the untreated cells. There is a significant difference between the two groups. The results show that the DIST NPs entered the cells through endocytosis and gradually released the drugs in the cytoplasm.

### Tumor Inhibition Effect of the Dual-Mode Imaging and PTT/Chemo Synergistic Theranostic Nanoparticles *In Vitro*

**Figure 4:**
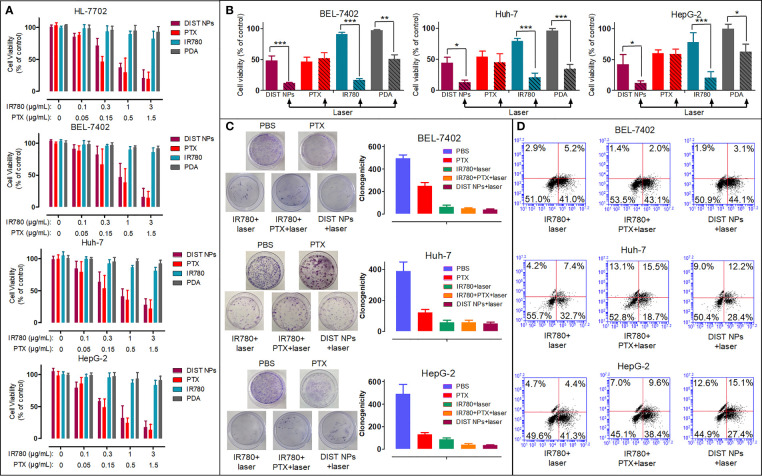
The *in vitro* cell inhibition results of the DIST NPs in Huh-7, BEL-7402, HepG2, and HL-7702 cells (n = 3) **(A)**. The *in vitro* PTT effect of the DIST NPs and their main components (n = 3) **(B)**. The colony formation test results of the DIST NPs and their main components (n = 3) **(C)**. The flow cytometry results **(D)**. The * represents p < 0.05; **p < 0.01; and ***p < 0.001.

The CCK-8 assay results are shown in **Figure 4A**. PDA and IR780 showed no significant toxicity in all four cell lines. The inhibitory effects of PTX and DIST NPs in all four cell lines rose with the increase of their concentrations and with no significant difference between them. The results showed that the DIST NPs effectively released the loaded PTX after entering the cells, performing a cell inhibitory effect. The PTT was introduced in the later CCK-8 assay (**Figure 4B**). With the 808-nm laser irradiation, the DIST NPs, IR780 and PDA groups showed apparent cell inhibitory effects. It is worth noting that the cell survival rate of the DIST NPs treatment group was lower than those of the IR780 + laser irradiation group and PTX treatment group. These results fully prove that the DIST NPs can effectively play the synergistic effect of PTT and chemotherapy in cells. The colony formation assay results (**Figure 4C**) further prove that the DIST NPs had excellent synergistic therapeutic effects. There was no significant difference in the numbers of clones between the DIST NPs group and the PPT + IR780 + laser irradiation group. The results of flow cytometry analysis (**Figure 4D**) show that the DIST NPs can effectively induce cell apoptosis, of which, the therapeutic effect is better than that of chemotherapy or PTT alone.

### Dual-Mode Imaging and PTT/Chemo Synergistic Theranostic Nanoparticles *In Vivo* Toxicity

**Figure 5:**
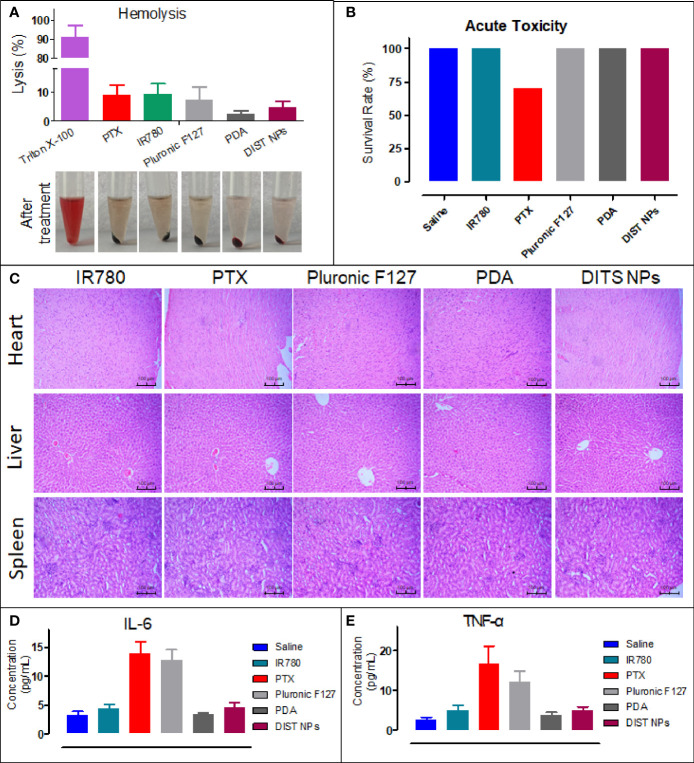
*In vivo* toxicity evaluation of the DIST NPs. The hemolysis test results of the DIST NPs. Pluronic F127 is the main surfactant in preparation of the DIST NPs. Therefore, the toxicity should be evaluated (n = 3) **(A)**. The *in vivo* acute toxicity test results of the DIST NPs on the BALB/c mice **(B)**. The pathological analysis of the DIST NPs and the main components to the heart, liver, and kidney **(C)**. The effects of the DIST NPs and its components to the main inflammatory factors *in vivo* (n = 4) **(D, E)**.

Before testing the *in vivo* imaging and antitumor effect, the *in vivo* safety of the DIST NPs needs to be evaluated. It is notable to point at whether the DIST NPs and their main components would cause hemolysis after intravenous injection. The results are shown in **Figure 5A**. The DIST NPs and their main components, PDA, IR780 PTX, and Pluronic F127, did not cause a significant erythrocyte rupture, while the hemolysis rate of the positive control group was 90%. The results show that the DIST NPs have an injection safety. The acute toxicity of the DIST NPs was evaluated by BALB/c mice. As shown in **Figure 5B**, within 14 d, no mouse died in the DIST NPs-treated group, and no adverse symptoms such as convulsion, depression, and appetite loss were found in mice except a few mice in the PTX-treated group. The survival statuses of mice in the PDA- and IR780-treated groups were also acceptable. Only three mice in the PTX-treated group died, and some mice had symptoms such as anorexia, weight loss, and mental malaise. In the pathological analysis of the mice organs (**Figure 5C**), the DIST NPs group mice only had a little lymphocyte infiltration in the liver, and no significant pathological changes were found in the other main organs. The blood concentration of two inflammatory factors, IL-6 and TNF-α, were measured (**Figures 5D, E**). Only two inflammatory factors of the PTX- and Pluronic F127-treated mice increased to a certain level, and the other groups, including a control group treated with normal saline, maintained a low inflammatory factors concentration. These results prove that the DIST NPs have a good biocompatibility *in vivo* and can be safely applied.

### Dual-Mode Imaging and PTT/Chemo Synergistic Theranostic Nanoparticles *In Vivo* Imaging

**Figure 6:**
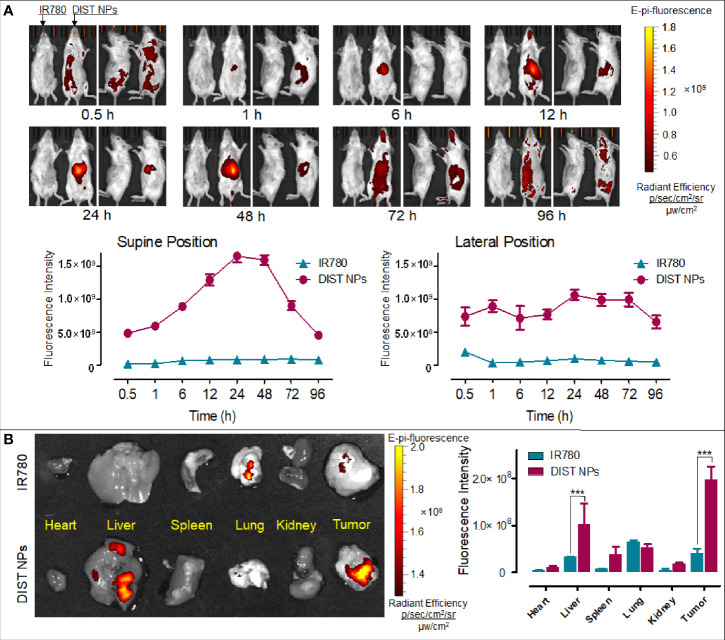
The *in vivo* NIFI effects of the DIST NPs. The dynamic distributions of the NIF signal distributions of IR780 and DIST NPs in the orthotopic hepatic carcinoma model mice **(A)**. The photographs were taken in supine position (left) and lateral position (right), respectively. Within each position, the left photo is the IR780-treated mouse and the right one is the DIST NPs-treated one. The residual fluorescent signals and their quantitative results in the organs and tumors of the mice **(B)**. The data of signal quantitation were extracted at more than three areas. The *** represents p < 0.001.

Firstly, the NIFI of the DIST NPs was evaluated on the orthotopic hepatic carcinoma model mice. As **Figure 6A** shows, in the DIST NPs-injected mice, the fluorescent signals began to accumulate in the abdomen 1 h after injection. The fluorescent intensity increased continuously over time, reached its highest at 24 h, and maintained through to 48 h, then decreased slowly. The fluorescent signal in the tumor was maintained until 72 h, showing an excellent *in vivo* NIFI effect. Moreover, the result also demonstrated that the DIST NPs could significantly prolong the internal circulation time of IR780. This property is crucial for the application of *in vivo* monitoring. After euthanasia, the residual signals in the main organs and tumors were detected (**Figure 6B**). There are only weak signals in the lungs and tumors of the mouse injected with IR780. In contrast, the DIST NPs-treated mice have apparent signals in the tumors and livers. However, the signals of metastatic lesions and spleen are relatively low. The results show that the DIST NPs can prolong the circulation time of drugs and can be effectively enriched in liver tumors, showing a good NIFI ability.

**Figure 7:**
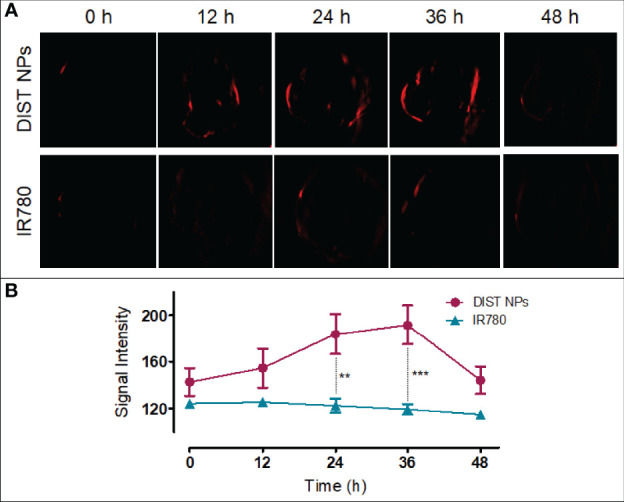
The *in vivo* PAI results of the DIST NPs. The dynamic distributions of PA signal **(A)** and quantitative analysis of PA signal in the double-tumor mice **(B)**. The ** represents p < 0.01; and ***p < 0.001.

PAI results are shown in **Figure 7A**. After injection, no obvious PA signal was found in the tumor area of the two mice. Later, the PA signal of the IR780-treated mice was consistently weak. In contrast, the PA signal of the DIST NPs-treated mice gradually gathered in the tumor over time. At 12 h, an obvious PA signal was detected in the tumor. At 24 h, the tumor PA signal was further enhanced. The PA signal reached its highest point at 36 h and began to drop gradually after that. At 48 h, there was still a weak signal in the tumor area. In this case, the PA signal did not only originate from IR780, but also came from PDA. The PDA was gradually degraded in the tumor tissue; therefore, the weakening of the signal was consequent on the decomposition of PDA. The quantitative results of the PA signal intensity are shown in **Figure 7B**. The PA signal changing trends of the two groups were significantly different. The signal of the DIST NPs group is much higher than that of the free IR780 group, even the PDA has been degraded. The results show that the DIST NPs can effectively realize tumor PAI.

### Antitumor Effect of CNC Nanoparticles *In Vivo*

**Figure 8:**
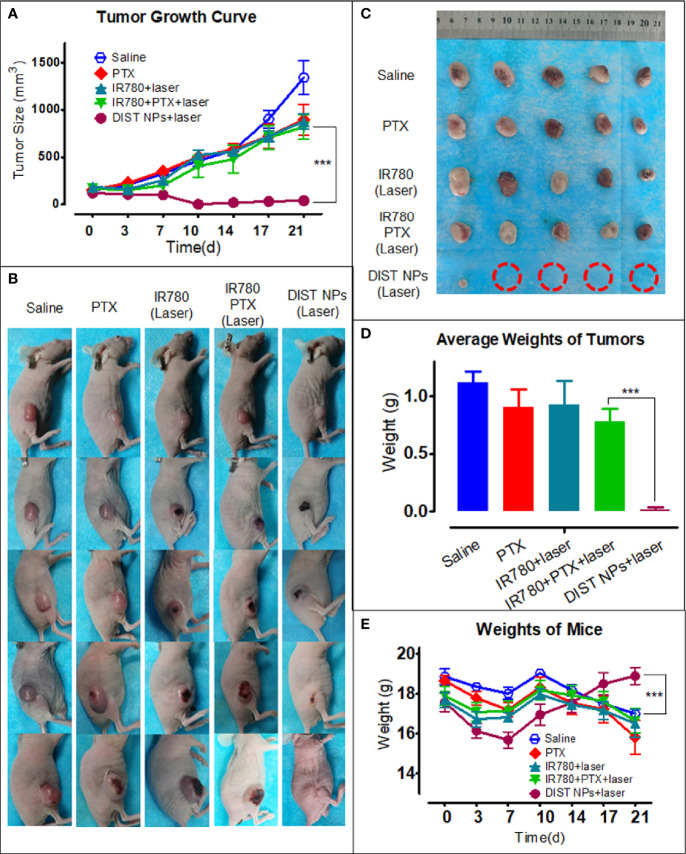
The antitumor effect of the DIST NPs *in vivo*. The tumor volume growth curve of each group (n = 5) **(A)**. The photographs of all the mice in the tumor treatment experiment **(B)**. The photographs of the residual tumors taken out after the treatment. The red circles represent the ablated tumors **(C)**. The tumor weighing results (n = 5) **(D)**. The mean weight changing curve of the mice in the experiment (n = 5) **(E)**. The *** represents p < 0.001.

The antitumor effect of DIST NPs *in vivo* was evaluated by the subcutaneous tumor model of hepatic carcinoma. **Figure 8A** shows the tumor growth curve. The graph indicates that the free PTX-treated mice did not show a significant antitumor effect at the dose of 1 mg/kg. IR780 also failed to inhibit tumor growth even with the 808-nm laser irradiation assistance, leaving only a few burning marks on some tumors. A similar situation prevails in the chemotherapy + PTT combined treatment group, there was no apparent inhibitory effect either. No significant difference in the tumor growth rate was found between the above three groups and the normal saline group. In contrast, the DIST NPs group showed an excellent antitumor effect. The tumors of four of the five mice were completely ablated. The average tumor volume was close to the lowest value after 10 d. **Figure 8B** shows the photographs of all the mice during the treatment. The tumors of mice in the DIST NPs group showed burning signs very early. After the scab, the treated area had recovered. However, the tumors of the other four groups kept on growing. **Figure 8C** shows the photographs of all the tumors taken out of the mice after euthanasia. Only very tiny tumors were found in the DIST NPs group. There is no significant tumor volume difference between the other four groups. The tumor weight results (**Figure 8D**) also show the same scenario. In addition, the average body weight of mice in the DIST NPs group was decreased in the first week, and then it continuously increased until the end of the experiment. However, the bodyweight of mice in the other groups decreased significantly (**Figure 8E**). These results further prove the therapeutic effect and *in vivo* safety of the DIST NPs.

## Discussion

Based on the estimation of the WHO, more than 1 million patients worldwide will die of hepatic carcinoma in the year 2030. Currently, surgical resection is still the primary treatment for hepatic carcinoma. However, hepatic carcinoma is more likely transformed from hepatitis and liver cirrhosis. Most liver tumors are in their middle- and late-stage when diagnosed, and less than 30% of them can be removed by surgical resection. Those patients who received surgeries still have a recurrence and metastasis rate of more than 70% within five years (37–39). Image-guided tumor ablation is the most commonly used treatment in the clinic (37, 40). PAI, a new molecular imaging technology, has the advantages of being non-invasive and non-ionized. Meanwhile, high selection and high penetration depth make PAI capable of tracking the occurrence and development of various tumors. Hence, PAI is suitable for tumor diagnosis and treatment monitoring (41, 42). It generates heat during the operation and is naturally related to PTT. The two can be combined to establish an integrated diagnosis and treatment system. Both melanin and NIF dyes can be used as molecular probes for PAI, and they also have a PTT effect. PTT is often used in combination with chemotherapy to achieve the effect of a synergistic treatment. Therefore, a multifunctional nanocarrier with NIFI/PAI optical dual-mode imaging and PTT/chemotherapy combined therapeutic functions was constructed in this study. With PDA as the encapsulation material, the NIF dye IR780 with photothermal effect, and the chemotherapy molecule PTX were wrapped in a nanoparticle. Those functional molecules can be delivered to tumor tissues synchronously, achieving the effects of long-term circulation, high bioavailability, and low toxic and side effects. The DIST NPs have a compact spherical structure, smooth and weak negative charged surface, and a size of approximately 100 nm. DIST NPs have good mono-dispersity and colloidal stability in various physiological solution environments. There is neither agglutination nor precipitation in the long-term observation, and their overall particle size remains well. DIST NPs stably loaded the drugs with only a small amount of release in the neutral pH environment. When the temperature rises or the pH decreases, the DIST NPs will decompose and gradually release the payloads. In the subsequent cell experiments, the result preliminarily proved that the DIST NPs enter the hepatoma cells by active transportation (endocytosis). These properties ensure that the DIST NPs can effectively tackle the clearence after entering the body. This feature improves their stability and prolongs their circulation time in the body, increasing the dose of drugs delivered to the tumor by NPs through the EPR effect. After that, PTT and chemotherapy effects of the DIST NPs were demonstrated. Based on the results above, we consider that the DIST NPs could perform the NIFI/PAI optical dual-mode imaging and PTT/chemotherapy combined therapeutic functions.

The purpose of the diagnosis and treatment integration is to realize the effect of diagnosis adjuvant treatment and the synchronous diagnosis with therapy (9). Therefore, an accurate diagnosis method is fundamental in the whole system. The imaging method is the most intuitive and reliable method for tumor diagnosis. During the PAI process, the laser pulses are guided to a specific tissue site. The contrast agents enriched in the site absorb energy, generate heat in surrounding tissues through vibration relaxation, and generate pressure waves and ultrasonic signals (43, 44). This technology depends on endogenous or exogenous PA signal molecules. Nanomaterials can be equipped with unique physical and chemical properties such as light, heat, magnetism, electricity, and chemistry. Many nanomaterials have been used in PAI (45–49). As an alliance in imaging, fluorescence imaging has become one of the most effective technologies in life system monitoring with high timeliness (50). NIF has become an essential method in fluorescence imaging because of its good tissue penetration, low tissue absorption, and low cost (16). As a NIF dye, IR780 has a good photothermal effect and can be used for NIFI/PAI dual-mode imaging. The coating material PDA also has characteristics similar to melanin and can also be used for PAI. These ingredients ensure the NIFI/PAI dual-mode imaging function of DIST NPs. The *in vivo* imaging effect of the DIST NPs was verified in the hepatic carcinoma model mice. In the orthotopic hepatic carcinoma model mice, DIST NPs showed an excellent tumor tissue accumulation ability. The signals were detected in the abdominal tumor area only one hour after injection and gradually increased over time. The signals remained at a high level from 12 to 48 h and could still be detected until 72 h. DIST NPs considerably prolonged the *in vivo* circulation time and tumor enrichment of IR780 and played an excellent tumor NIFI effect compared with the directly injected free dye. The PAI results showed that, compared with free IR780, the DIST NPs have obvious PA signals in the tumor area. The high-intensity signals were maintained for more than 36 h after injection. These results fully prove that the DIST NPs can perform NIFI/PAI dual-mode imaging.

Finally, the *in vivo* toxicity and antitumor effect of the DIST NPs were evaluated in the tumor model mice. As intravenously administered nanoparticles, DIST NPs should not influence the red blood cells. Through the hemolysis experiment results, DIST NPs has been proven to possess a good injection safety. The DIST NPs did not cause any apparent hemolysis. The hemolysis rate of the components of the DIST NPs did not exceed 5%. Later in the acute toxicity experiment, DIST NPs did not cause mouse death and obvious organ pathological changes, showing a good biocompatibility. PDA and IR780 did not show an apparent toxicity during the experiment. Only the mice in the PTX treatment group died. However, the overall survival rate reached 70% due to the low PTX dose. Pathological and inflammatory factor analysis results also confirmed the low toxicity of the DIST NPs. Overall, the DIST NPs have a good biosafety. DIST NPs showed excellent tumor intervention effects *in vivo*. In the experiment, PTX and IR780 in all treatment groups were 1 mg/kg and 2 mg/kg, respectively. Such doses had almost no significant therapeutic effect in the other three free drug control groups but had a significant tumor inhibition in the DIST NPs group. Until the end of the experiment, four out of five mice had their tumors completely ablated. A relatively small tumor remained in only one mouse. The weight and physiological status of the DIST NPs-treated mice were also significantly better than those of the other groups. These results fully prove that the DIST NPs can effectively exert the synergistic effect of PTT/chemotherapy *in vivo*.

## Conclusion

Aiming at the shortcomings of the clinical diagnosis and treatment of hepatic carcinoma, according to the concept of theranostics, we designed and prepared the DIST NPs, multifunctional nanoparticles with NIFI/PAI dual-mode imaging, and PTT/chemotherapy functions. The delivery, imaging and anti-hepatic carcinoma effects of the DIST NPs were evaluated *in vivo* and *in vitro*. The results show that the DIST NPs fully achieved the original goals. At the cellular level, they can enter the hepatoma cells through endocytosis and release the loaded active molecules, realizing the synergistic PTT and chemotherapy effect. In the mice model, DIST NPs did not show apparent toxic and side effects. DIST NPs showed excellent NIFI and PAI effects. The *in vivo* signals were strong and lasted for a long time. The DIST NPs still showed an excellent *in vivo* antitumor effect and ablated most tumors at a low dose level. This study provides experimental evidence for the development of a new treatment for hepatic carcinoma.

## Data Availability Statement

The raw data supporting the conclusions of this article will be made available by the authors, without undue reservation.

## Ethics Statement

The animal study was reviewed and approved by the Laboratory Animal Administration Committee of Xi’an Medical University.

## Author Contributions

YZ, KL, CH, and RJ designed the study. YZ, BL, YFZ, and ZS performed the experiments. YZ, BL, KL, CH, and RJ analyzed the results and data. YZ, BL, and CH prepared the manuscript. YZ, CH, and JR modified the manuscript. All authors contributed to the article and approved the submitted version.

## Funding

This study was supported, in part, by the National Natural Science Foundation of China (81801863, 82102832), Innovation Capability Support Program of the Shaanxi Province (2020KJXX-050), the Research and Development Program of Innovation Chain for Key industries in Shaanxi Province (Program No. 2021ZDLSF02-09), Research Foundation of China-Nepal Friendship Research Center of RJ (18LJM03), and the Institutional Foundation of the First affiliated hospital of Xi’an Jiaotong University (2019QN-08).

## Conflict of Interest

The authors declare that the research was conducted in the absence of any commercial or financial relationships that could be construed as a potential conflict of interest.

## Publisher’s Note

All claims expressed in this article are solely those of the authors and do not necessarily represent those of their affiliated organizations, or those of the publisher, the editors and the reviewers. Any product that may be evaluated in this article, or claim that may be made by its manufacturer, is not guaranteed or endorsed by the publisher.
